# Lung Function and Incidence of Chronic Obstructive Pulmonary Disease after Improved Cooking Fuels and Kitchen Ventilation: A 9-Year Prospective Cohort Study

**DOI:** 10.1371/journal.pmed.1001621

**Published:** 2014-03-25

**Authors:** Yumin Zhou, Yimin Zou, Xiaochen Li, Shuyun Chen, Zhuxiang Zhao, Fang He, Weifeng Zou, Qiuping Luo, Wenxi Li, Yiling Pan, Xiaoliang Deng, Xiaoping Wang, Rong Qiu, Shiliang Liu, Jingping Zheng, Nanshan Zhong, Pixin Ran

**Affiliations:** 1 State Key Laboratory of Respiratory Disease, Guangzhou Institute of Respiratory Diseases, First Affiliated Hospital, Guangzhou Medical University, Guangzhou, Guangdong, China; 2 First Municipal People Hospital of Shaoguan, Shaoguan, Guangdong, China; 3 Third Affiliated Hospital, Guangzhou Medical University, Guangzhou, Guangdong, China; Simon Fraser University, Canada

## Abstract

**Background:**

Biomass smoke is associated with the risk of chronic obstructive pulmonary disease (COPD), but few studies have elaborated approaches to reduce the risk of COPD from biomass burning. The purpose of this study was to determine whether improved cooking fuels and ventilation have effects on pulmonary function and the incidence of COPD.

**Methods and Findings:**

A 9-y prospective cohort study was conducted among 996 eligible participants aged at least 40 y from November 1, 2002, through November 30, 2011, in 12 villages in southern China. Interventions were implemented starting in 2002 to improve kitchen ventilation (by providing support and instruction for improving biomass stoves or installing exhaust fans) and to promote the use of clean fuels (i.e., biogas) instead of biomass for cooking (by providing support and instruction for installing household biogas digesters); questionnaire interviews and spirometry tests were performed in 2005, 2008, and 2011. That the interventions improved air quality was confirmed via measurements of indoor air pollutants (i.e., SO_2_, CO, CO_2_, NO_2_, and particulate matter with an aerodynamic diameter of 10 µm or less) in a randomly selected subset of the participants' homes. Annual declines in lung function and COPD incidence were compared between those who took up one, both, or neither of the interventions.

Use of clean fuels and improved ventilation were associated with a reduced decline in forced expiratory volume in 1 s (FEV_1_): decline in FEV_1_ was reduced by 12 ml/y (95% CI, 4 to 20 ml/y) and 13 ml/y (95% CI, 4 to 23 ml/y) in those who used clean fuels and improved ventilation, respectively, compared to those who took up neither intervention, after adjustment for confounders. The combined improvements of use of clean fuels and improved ventilation had the greatest favorable effects on the decline in FEV_1_, with a slowing of 16 ml/y (95% CI, 9 to 23 ml/y). The longer the duration of improved fuel use and ventilation, the greater the benefits in slowing the decline of FEV_1_ (*p*<0.05). The reduction in the risk of COPD was unequivocal after the fuel and ventilation improvements, with an odds ratio of 0.28 (95% CI, 0.11 to 0.73) for both improvements.

**Conclusions:**

Replacing biomass with biogas for cooking and improving kitchen ventilation are associated with a reduced decline in FEV_1_ and risk of COPD.

**Trial Registration:**

Chinese Clinical Trial Register ChiCTR-OCH-12002398

*Please see later in the article for the Editors' Summary*

## Abstract 2

Pixin Ran, Nanshan Zhong, and colleagues report that cleaner cooking fuels and improved ventilation were associated with better lung function and reduced COPD among a cohort of villagers in Southern China.

*Please see later in the article for the Editors' Summary*

## Editors' Summary

**Background:**

Nearly 3 billion people in developing countries heat their homes and cook by burning biomass—wood, crop waste, and animal dung—in open fires and leaky stoves. Burning biomass this way releases pollutants into the home that impair lung function and that are responsible for more than a million deaths from chronic obstructive pulmonary disease (COPD) every year. COPD is a group of diseases that interfere with breathing. Normally, air is breathed in through the nose or mouth and travels down the windpipe into two bronchial tubes (airways) in the lungs. These tubes branch into smaller tubes (bronchioles) that end in bunches of tiny air sacs (alveoli). Oxygen in the air passes through the thin walls of these sacs into small blood vessels and is taken to the heart for circulation round the body. The two main types of COPD—chronic bronchitis (long-term irritation and swelling of the bronchial tubes) and emphysema (damage to the walls of the alveoli)—make it hard for people to breathe. Most people with COPD have both chronic bronchitis and emphysema, both of which are caused by long-term exposure to cigarette smoke, indoor air pollution, and other lung irritants. Symptoms of COPD include breathlessness during exercise and a persistent cough that produces large amounts of phlegm (mucus). There is no cure for COPD, but drugs and oxygen therapy can relieve its symptoms, and avoiding lung irritants can slow disease progression.

**Why Was This Study Done?:**

Exposure to indoor air pollution has been associated with impaired lung function and COPD in several studies. However, few studies have assessed the long-term effects on lung function and on the incidence of COPD (the proportion of a population that develops COPD each year) of replacing biomass with biogas (a clean fuel produced by bacterial digestion of biodegradable materials) for cooking and heating, or of improving kitchen ventilation during cooking. Here, the researchers undertook a nine-year prospective cohort study in rural southern China to investigate whether these interventions are associated with any effects on lung function and on the incidence of COPD. A prospective cohort study enrolls a group of people, determines their characteristics at baseline, and follows them over time to see whether specific characteristic are associated with specific outcomes.

**What Did the Researchers Do and Find?:**

The researchers offered nearly 1,000 people living in 12 villages in southern China access to biogas and to improved kitchen ventilation. All the participants, who adopted these interventions according to personal preferences, completed a questionnaire about their smoking habits and occupational exposure to pollutants and had their lung function measured using a spirometry test at the start and end of the study. Some participants also completed a questionnaire and had their lung function measured three and six years into the study. Finally, the researchers measured levels of indoor air pollution in a randomly selected subset of homes at the end of the study to confirm that the interventions had reduced indoor air pollution. Compared with non-use, the use of clean fuels and of improved ventilation were both associated with a reduction in the decline in lung function over time after adjusting for known characteristics that affect lung function, such as smoking. The use of both interventions reduced the decline in lung function more markedly than either intervention alone, and the benefits of using the interventions increased with length of use. Notably, the combined use of both interventions reduced the risk of COPD occurrence among the study participants.

**What Do These Findings Mean?:**

These findings suggest that, among people living in rural southern China, the combined interventions of use of biogas instead of biomass and improved kitchen ventilation were associated with a reduced decline in lung function over time and with a reduced risk of COPD. Because participants were not randomly allocated to intervention groups, the people who adopted the interventions may have shared other unknown characteristics (confounders) that affected their lung function (for example, having a healthier lifestyle). Thus, it is not possible to conclude that either intervention actually caused a reduction in the decline in lung function. Nevertheless, these findings suggest that the use of biogas as a substitute for biomass for cooking and heating and improvements in kitchen ventilation might lead to a reduction in the global burden of COPD associated with biomass smoke.

**Additional Information:**

Please access these websites via the online version of this summary at http://dx.doi.org/10.1371/journal.pmed.1001621.

- The US National Heart, Lung, and Blood Institute provides detailed information for the public about COPD
- The US Centers for Disease Control and Prevention provides information about COPD and links to other resources (in English and Spanish)
- The UK National Health Service Choices website provides information for patients and carers about COPD, personal stories, and links to other resources
- The British Lung Foundation, a not-for-profit organization, provides information about COPD in several languages
- The Global Initiative for Chronic Obstructive Lung Disease works to improve prevention and treatment of COPD around the world
- The World Health Organization provides information about all aspects of indoor air pollution and health (in English, French, and Spanish)
- MedlinePlus provides links to other information about COPD (in English and Spanish)

## Introduction

Biomass, including wood, agricultural residues, charcoal and dung, is widely used for cooking in developing countries [1],[2]. A number of pollutants, such as carbon monoxide (CO), particulate matter, sulfur dioxide (SO_2_), and nitrogen dioxide (NO_2_), are produced from burning biomass, leading to indoor air pollution. Such air pollution has been reported to be associated with an increased risk of chronic obstructive pulmonary disease (COPD), respiratory symptoms, and impaired lung function in a number of both cross-sectional and case-control studies [3]–[10]. However, few studies have assessed the long-term effects of the replacement of biomass fuel and the improvement of kitchen ventilation during cooking on COPD and longitudinal lung function [11]–[13]. Thus, we conducted a 9-y prospective cohort study (2002–2011) in southern China.

## Methods

### Study Design

In this 9-y prospective cohort study, 996 participants aged at least 40 y were offered cooking interventions (i.e., the opportunity to use clean fuels [biogas] and to have improved kitchen ventilation) with the support of local village committees beginning in November 2002. Participants adopted the interventions according to their preference. The participants underwent spirometry tests and questionnaire interviews once every 3 y to assess association of the adoption of these non-randomized interventions with the subsequent rate of lung function decline and the incidence of COPD.

### Ethical Statement

Our study protocol was approved by the Medical Ethics Committee of Guangzhou Institute of Respiratory Diseases on May 20, 2002. A written informed consent form was signed by all participants prior to the study's start, and the whole study was conducted in accordance with the principles expressed in the Declaration of Helsinki.

### Study Site and Population

This study was conducted in Yunyan, which is a rural area in southern China with a population of approximately 14,000. In this region, there is no major industry and little motorized transportation, annual income per person in 2003 was US$455.23, and the houses rarely require heating because of the warm climate. Prior to 2002, biomass was the major fuel for most households in this area, and cooking was predominately performed indoors with an open-fire traditional cooking stove in a small adobe kitchen with a thatched or tile roof and without ventilation facilities.

Based on a cross-sectional survey of COPD conducted in this region in 2002 [3], a total of 996 participants (one person per household) in 12 villages who met the inclusion criteria were invited to participate in this study. The inclusion criteria included being at least 40 y of age, cooking with biomass (for at least 1 h per day for more than 6 mo) with poor ventilation (i.e., no ventilation facility such as an exhaust fan or chimney in the kitchen), having completed an acceptable baseline survey, and having given written informed consent to participate in this cohort study. The exclusion criteria included a diagnosis of active tuberculosis, asthma, obvious bronchiectasis, cystic fibrosis, interstitial lung disease, pulmonary thromboembolic disease, or malignant tumor; a history of thoracotomy with pulmonary resection, uncontrolled or serious diseases, or other symptoms that could potentially affect the spirometry test; or a plan to move out of the area permanently. A participant was considered lost to follow-up if we could not contact the participant; if he or she had moved to other place, died, withdrawn consent, or refused to proceed; or if he/she was unable to continue on the study for any reason.

### Interventions

The interventions included use of clean fuels (i.e., providing support and instruction for installing household biogas digesters, to allow participant to use biogas as a cooking fuel) and improved kitchen ventilation (i.e., providing support and instruction for improving biomass stoves and/or installing exhaust fans). In contrast with traditional stoves, the improved biomass stoves that were installed had a chimney, an air chute, and a surplus heat recovery system (Figure S1); these new stoves cost US$49.02–US$81.70. Biogas is combustible gas produced via the breakdown of biomass at atmospheric temperature using anaerobic digestion. Building a household biogas digester with a gas production rate >0.2 m^3^ per cubic meter of digester volume per day usually costs about US$326.80. These interventions were offered to all participants through the local village committees' organization and implementation channels. The participants adopted the interventions (i.e., use of clean fuels, improved ventilation, both, or neither) according to their own choice. To reduce any differences due to education level or socioeconomic status in the understanding of intervention benefits and in the skills involved in changing stoves and building household biogas digesters [14], education courses were given to all participants through lectures, bulletins, posters, manuals, and consultation. In addition, both technical assistance and partial financial aid were provided by the local government to the residents who adopted the interventions. The amount of financial aid was determined by intervention type, e.g., US$16.34–US$32.68 for improving ventilation and US$163.40 for building a household biogas digester; this financial aid was given to those who were in need, or as an incentive for those who would otherwise not take up the intervention. Intervention implementation and education were performed once every 3 mo in the first year, and thereafter once a year throughout the study.

### Follow-Up and Outcome Measures

Detailed questionnaire interviews and spirometry tests were performed for all participants at the first (2002) and the last visit (2011). In 2005 and 2008, spirometry tests were conducted for a portion of the participants, and brief questionnaire interviews were conducted for all participants. At the end of the study, CO concentration in the exhaled gas of each participant was measured, and indoor air pollutants (i.e., SO_2_, CO, CO_2_, NO_2_, and particulate matter with an aerodynamic diameter of 10 µm or less [PM_10_]) were measured for one-third of the participants' homes, which were randomly selected through a systematic random process as previously described for the baseline survey [3]. That use of improved cooking fuels and improved kitchen ventilation lowered air pollution, and that self-reported ex-smokers had ceased smoking, was confirmed using a combination of home visits, exhaled gas CO concentrations of the individuals, and measures of indoor air pollutants at the end of the follow-up. Annual decline in lung function and COPD incidence were compared between the intervention groups.

#### Questionnaires

The questionnaire from the COPD Epidemiological Survey in China was used in our study [3],[7],[8],[15]. The detailed questionnaire administered at the beginning and end of the study included demographic characteristics, socioeconomic status indicators (education level, occupation, living area size per person, and self-reported economic status), respiratory symptoms, and risk factors (occupational exposure to dust/gases/fumes, cigarette smoking, exposure to environmental tobacco smoke (ETS), kitchen cooking fuels, and ventilation status). The brief questionnaire administered in 2005 and 2008 included change of risk factors and respiratory symptoms.

We categorized the participants' smoking status at each visit as never smoker, ex-smoker, or current smoker [15]. Participants who had smoked for less than 6 mo or who had smoked fewer than 100 cigarettes in their lifetime were defined as never smokers [8],[15]. Current smokers were those who smoked tobacco products at the time of survey and included continuous and intermittent smokers, those who had quit but resumed or relapsed, those who had quit less than 6 mo ago. Ex-smokers were those who had not smoked tobacco products for the last 6 mo or longer at the time of survey. Smoking cessation in ex-smokers was confirmed by exhaled CO<7 parts per billion; in participants with exhaled CO≥7 parts per billion, smoking cessation was confirmed by contacting their family members or neighbors.

ETS exposure was assessed by asking participants whether they could smell tobacco smoke at home or at work for at least 1 h a day [15]. We classified ETS exposure as improved (i.e., no exposure or decreased exposure from baseline) or not improved (no change or increased exposure from baseline) [15]. Occupational exposure to dust/gases/fumes was defined as exposure for more than 1 y in a participant's lifetime [8].

We defined “clean fuel use” as having used biogas, liquefied petroleum gas, or electricity for cooking for at least 1 h per day for more than 6 mo during the study period, and “improved ventilation” as having had an improved stove and/or exhaust fan during cooking for at least 6 mo according to a combination of self-reporting and home visits. Kitchen ventilation and cooking fuels were assessed during home visits through observing the current cooking apparatus, the degree of discoloration of the kitchen walls, and the type of fuels and by asking about the duration of kitchen use, the previous cooking apparatus, and recent cleaning. Intervention status was classified into four groups based on “clean fuel use” and “improved ventilation”: Group Neither (neither improved ventilation nor use of clean fuels), Group V-only (improved ventilation only), Group CF-only (use of clean fuels only), and Group Both (both improvements) (Figure S1). Intervention status was confirmed by home visits. To quantitatively assess the cumulative exposure to clean fuels during the study period, we used a “clean fuels index,” which was defined as the number of years of using clean fuels multiplied by the hours of cooking per day, similar to a “pack-year” for smoking intensity. The “biomass exposure index,” which captured baseline exposure to biomass fuels, was defined as the number of years of using biomass fuels before baseline multiplied by the hours of cooking per day.

#### Spirometry

Trained technicians performed spirometry in accordance with the criteria recommended by the European Respiratory Society [16],[17]. At least three acceptable and two reproducible measurements (i.e., the largest and second largest values for forced vital capacity [FVC] and forced expiratory volume in 1 s [FEV_1_] within 150 ml or 5%) that met the criteria were taken for each participant [17]. The largest values of FVC and FEV_1_ are reported. To minimize variation in the measurements from all tests over the 9 y as described above, spirometry was performed at almost the same time of a day (i.e., morning) for everyone each time, and measurements were performed in comparable seasons and temperature and humidity conditions as much as possible. The use of short- and long-acting bronchodilators was prohibited within 12 and 24 h before the test, respectively. A portable spirometer (Micro Medical) was used. For those with FEV_1_/FVC ratio <70%, a post-bronchodilator spirometry test was performed: this test was performed 15–20 min after the inhalation of 400 µg of salbutamol (Ventolin, GlaxoSmithKline) via a 500-ml spacer. We used 1993 reference values from the European Coal and Steel Community as our predicted values of FEV_1_; these values were already adjusted with conversion factors for Chinese people (i.e., male 0.95 and female 0.93) [18].

A diagnosis of COPD was defined by spirometry according to the diagnostic criteria of the Global Initiative for Chronic Obstructive Lung Disease [19], i.e., having a post-bronchodilator FEV_1_/FVC ratio <70%.

#### Indoor pollutants and exhaled CO measurement

All participants were tested twice for their exhaled CO concentration at the end of the study using a portable instrument (MicroCO, CareFusion) following its instructions. That is, at room temperature, the apparatus was opened, and, upon hearing a tone, the participant made a deep inspiration to maximum, and held his or her breath for a 20-s countdown until a green light turned on (indicating that screen display measurement was ready). The participant kept the oral device in his or her mouth and exhaled slowly until a value of CO concentration (parts per billion) registered and was recorded. The average value of two measurements for each participant was used in the analysis.

Indoor air pollutants were measured for one-third of the participants' homes selected by a systematic random sampling; indoor air pollutants were measured in the kitchen and other rooms during cooking using an automatic dust monitor (P-5L2C, Midwest Group) for PM_10_, an Interscan 4150 (Interscan) for NO_2_, an Interscan 4240 (Interscan) for SO_2_, and a TSI 7565 Q-TRAKTM (TSI) for CO and CO_2_. All air samples were taken 1.2–1.5 m above the floor of the house. Three measurements were obtained from different sampling sites that were 1 m away from the center of the cooking stove in the kitchen, and an average value was used for analysis. Three measurements were also obtained from different sampling sites in rooms that were located 3 m or further from the kitchen. Three outdoor measurements were obtained in the surrounding environment from different sampling sites outside the houses of study at the same time.

### Statistical Methods

The sample size was estimated according to an approximate standard deviation (SD) of the mean slope of the FEV_1_ value of 80 ml per year, a withdrawal rate of 30%, a two-sided type I error of 5%, and a power of 80% to detect a difference in an intervention response of 20 ml per year.

Participants who completed at least two spirometry tests comprising one at the start and another at the end of study could be included in the analysis. The measurements of air pollutants (after log transformation) and baseline characteristics were assessed using analysis of variance for continuous variables and a chi-squared test for categorical variables.

To analyze the longitudinal changes in lung function over time, we fitted mixed effects models using restricted (or residual) maximum likelihood after adjustment for confounders. This framework facilitates our use of all available data by “borrowing” information from earlier observed data on lung function to project later missing values under a missing-at-random assumption. At the same time, the uncertainty in these projections is accounted for in the calculation of standard errors (SEs) and test statistics. This analysis was implemented using the Proc Mixed procedure in SAS with a repeated statement.

In the final model, lung function level (e.g., FEV_1_) at each visit was the dependent variable; the different intervention groups were the specific fixed-effect independent variables; the baseline lung function level for that parameter (e.g., FEV_1_) was a covariate; and age, sex, education, smoking status and intensity, ETS exposure, COPD status, body mass index (BMI), occupational exposure to dust/gases/fumes, self-reported economic status, baseline biomass exposure index, the number of hours spent cooking each day, and living area size were fixed-effect confounding variables. The models also included interaction terms between time (defined by the variable “visit”) and intervention group, to allow for a possible time-varying effect. “Village” was not included in the final longitudinal lung function model because inclusion or exclusion of “village” in the model as a fixed effect did not affect the key effect estimates and showed no statistically significant difference between the two models in our earlier model selection process, using a likelihood ratio test and maximum likelihood estimation. Preliminary data analysis showed that the pattern of change in absolute values of lung function from baseline was approximately linear.

The selection of the appropriate type of covariance structure was accomplished by considering the biological features of the outcome variable and also by choosing the smallest Akaike's information criterion after fitting the models with alternative covariance structures. In the final models, an autoregressive order 1 structure covariance was chosen to account for the serial correlation of lung function within individuals, and an unstructured covariance was chosen to account for random variation in the intercept and slope parameters between communities and individuals. This analysis was repeated in the subgroups (i.e., men, women, non-smokers, and participants without COPD).

We used logistic regression modeling to estimate the odds ratio (OR) of COPD occurrence during the study period among participants without COPD at baseline, taking the above-mentioned potential confounders into account in the model; the variables sex, age, education, smoking intensity, self-reported economic status, clean fuel use, improved ventilation, and the number of hours spent cooking each day were in the model. The significance was set at *p*<0.05. All statistical analyses were performed using SAS version 9.1 software (SAS Institute).

## Results

### Description at Baseline and Follow-Up

**Figure 1:**
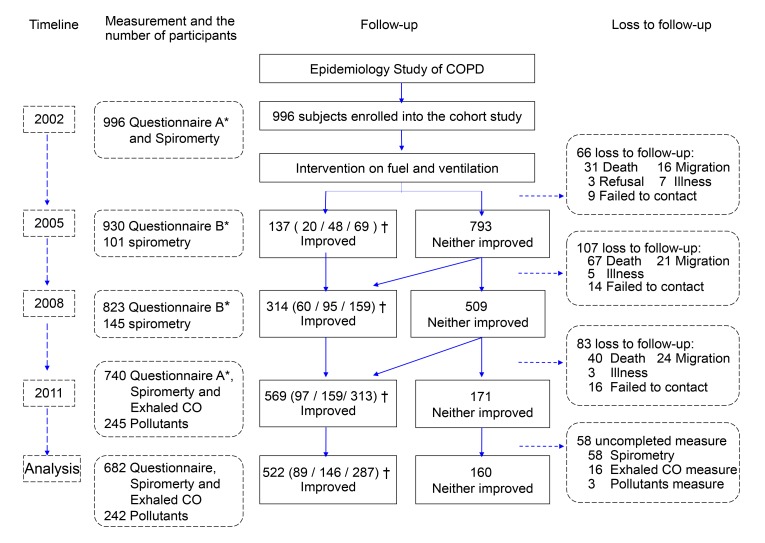
Study cohort flow chart. *Questionnaire A: detailed questionnaire interviews. Questionnaire B: brief questionnaire interviews. †Data given as the number of participants in Group V-only (improved ventilation only)/Group CF-only (use of clean fuels only)/Group Both, respectively.

Of 996 enrolled participants, 740 (74.3%) completed the follow-up examinations with questionnaires, and 724 (72.7%) completed the CO tests for exhaled gas; 682 (68.5%) had complete data for spirometry tests and were thus included in our analysis, and the homes of 242 participants were measured for indoor air pollutants. The reasons for loss to follow-up included migration, failure to contact, inability or ineligibility to continue, refusal, and death (Figure 1). The characteristics of included participants versus excluded ones are shown in Table S1. At baseline, there were no significant differences between the characteristics of the intervention groups except for the predicted values of FEV_1_ and FVC (Table 1).

**Table 1:** Characteristics of participants by status of fuel use and ventilation for cooking.

Time Point	Characteristic	Group				*p*-Value
		Neither (*n* = 160)	CF-only (*n* = 146)	V-only (*n* = 89)	Both (*n* = 287)	
**Baseline**	**Age, y**	55.5 (10.1)	54.8 (10.0)	54.7 (10.1)	53.4 (9.6)	0.17
	**Men, number (percent)**	75 (46.9)	73 (50.0)	38 (42.7)	125 (43.6)	0.57
	**Participants educated <6 y, number (percent)**	150 (93.8)	129 (88.4)	79 (88.8)	253 (88.2)	0.27
	**With COPD, number (percent)**	16 (10.0)	17 (11.6)	13 (14.6)	32 (11.1)	0.74
	**Occupational exposure to dust/gases/fumes, number (percent)** a	34 (21.1)	40 (27.6)	23 (25.8)	59 (20.7)	0.39
	**BMI, kg/m^2^**	22.4 (2.2)	22.6 (2.7)	22.2 (2.7)	22.8 (2.7)	0.24
	**Smoking intensity, pack-years** b	39.1 (30.4)	33.1 (19.9)	30.8 (24.3)	30.1 (20.5)	0.11
	**Current smoking status, number (percent)**					0.23
	Never smoker	95 (59.4)	84 (57.5)	55 (61.8)	183 (63.8)	
	Ex-smoker	5 (3.1)	13 (8.9)	4 (4.5)	11 (3.8)	
	Current smoker	60 (37.5)	49 (33.6)	30 (33.7)	93 (32.4)	
	**ETS exposure, number (percent)**	138 (86.3)	126 (86.3)	74 (83.1)	236 (82.2)	0.59
	**Self-reported poor economic status (net annual income per person<US$163.40/person), number (percent)**	12 (7.5)	12 (8.2)	5 (5.6)	15 (5.2)	0.60
	**Biomass exposure index** c	116.3 (69.4)	108.5 (73.1)	109.3 (78.8)	108.4 (62.8)	0.67
	**Living area size, m^2^/person**	13.2 (10.7)	13.2 (13.9)	10.2 (5.2)	13.4 (11.2)	0.10
	**FEV_1_, l**	2.20 (0.64)	2.24 (0.63)	2.09 (0.69)	2.20 (0.69)	0.43
	**Percent predicted FEV1**	97.1 (20.1)	97.0 (20.5)	89.8 (22.1)	93.3 (20.5)	0.016
	**FVC, l**	2.80 (0.74)	2.86 (0.70)	2.72 (0.77)	2.79 (0.78)	0.56
	**Percent predicted FVC**	103.7 (17.5)	104.3 (18.7)	98.3 (17.9)	100.1 (18.1)	0.015
	**FEV_1_/FVC ratio, percent**	78.3 (9.4)	78.0 (9.9)	76.4 (10.7)	78.4 (9.7)	0.36
**At the end of the study**	**Smoking intensity, pack-years** d	47.5 (31.2)	42.1 (21.6)	41.1 (27.0)	39.6 (22.0)	0.24
	**Current smoking status, number (percent)**					0.45
	Never smoker	93 (58.1)	84 (57.5)	55 (61.8)	181 (63.1)	
	Ex-smoker	14 (8.8)	22 (15.1)	8 (9.0)	29 (10.1)	
	Current smoker	53 (33.1)	40 (27.4)	26 (29.2)	77 (26.8)	
	**ETS exposure, number (percent)** e	118 (75.6)	116 (80.0)	66 (74.2)	221 (78.4)	0.68

Data are mean (SD) unless otherwise indicated. CF-only: only use of clean fuels; V-only: only improved ventilation; Both: both use of clean fuels and improved ventilation; Neither: neither use of clean fuels nor improved ventilation.

a There were nine participants whose data were missing, including two participants in Group Both, one participant in Group CF-only, and six participants in Group Neither.

b Calculated for 265 smokers (only one female), including 65 participants in Group Neither, 62 in Group CF-only, 34 in Group V-only, and 104 in Group Both.

c Biomass exposure index was defined as the number of years before baseline multiplied by the hours of exposure to biomass for cooking per day.

d Calculated for 269 smokers (only one female), including 67 participants in Group Neither, 62 in Group CF-only, 34 in Group V-only, and 106 in Group Both.

e There were ten participants whose data were missing, including five participants in Group Both, one participant in Group CF-only, and four participants in Group Neither.

Of the 682 participants with complete data for spirometry tests, at the end of the study, 287 (42.1%) were identified in Group Both, 160 (23.5%) in Group Neither, 146 (21.4%) in Group CF-only, and 89 (13.0%) in Group V-only (Table 1). 433 (63.5%) had used clean fuels for an average of 4.95 (SD 2.89) y since 2002, 249 (36.5%) still used polluting fuels (i.e., biomass fuel), 376 (55.1%) had improved ventilation in their kitchens (which they had had for an average 5.09 [SD 2.66] y), and 306 (44.9%) had unimproved ventilation (Table S2).

**Figure 2:**
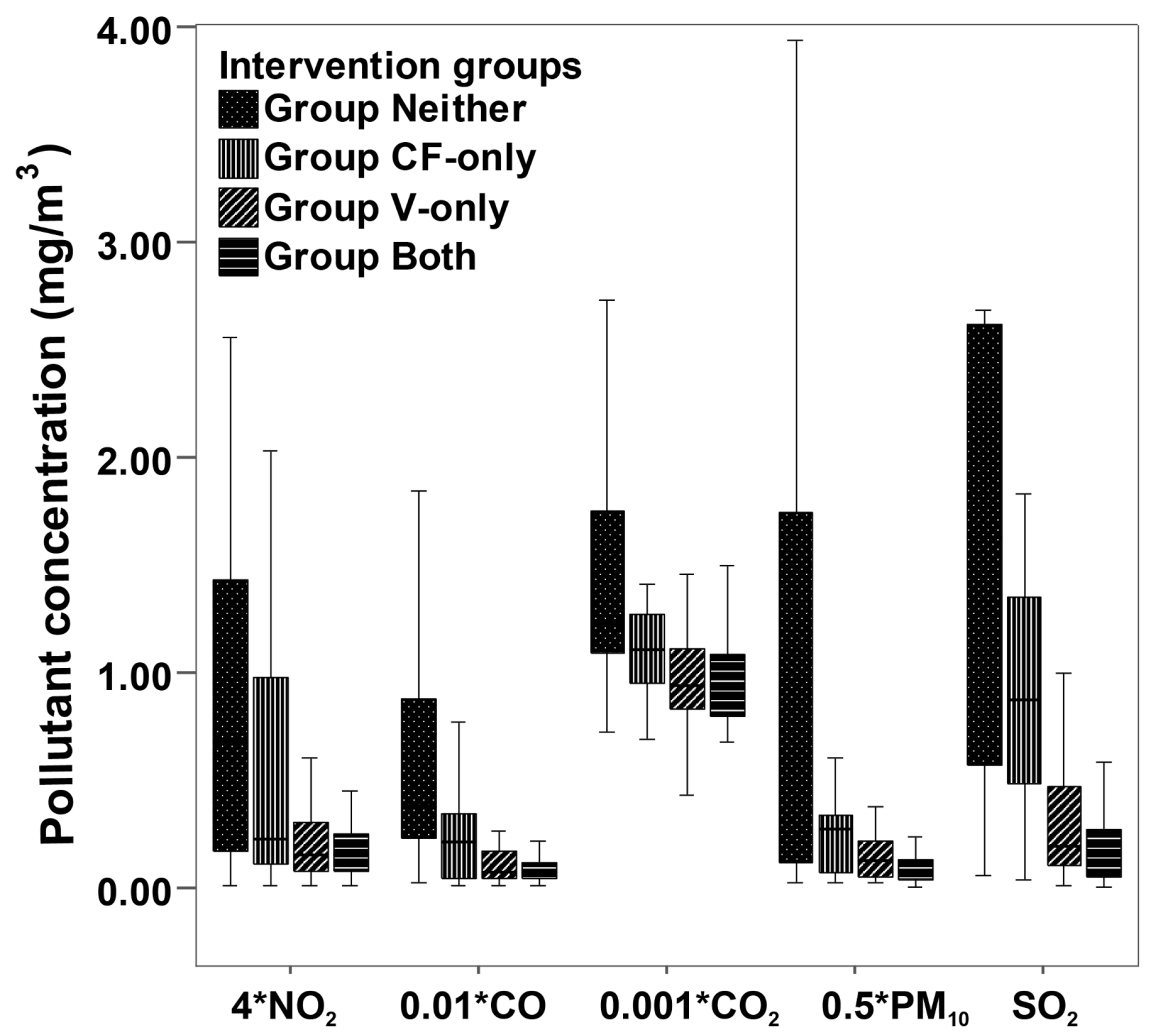
Box plots for indoor air pollutant concentrations in the kitchen during cooking, by intervention group. Data for pollutant concentration presented as median and 25th–75th percentiles. The total number of samples was 242: 67 in homes with neither improved ventilation nor clean cooking fuels, 28 in homes where clean fuels were used, 45 in homes with improved ventilation, and 102 in homes with both interventions. Pollutant concentrations are in milligrams per cubic meter for SO_2_, 0.5×milligrams per cubic meter for PM_10_, 0.01×milligrams per cubic meter for CO, 0.001×milligrams per cubic meter for CO_2_, and 4×milligrams per cubic meter for NO_2_. *p*<0.05 for comparison among groups.

The median concentrations of the pollutants CO, PM_10_, SO_2_, and NO_2_ in the kitchen were highest in Group Neither, and they were the lowest in Group Both, with a significant difference among the four groups at the end of the study (Figure 2), suggesting that self-reported intervention status was in agreement with the measurements.

### Lung Function

The average rate of annual decline in FEV_1_ was 18 ml/y (SE 3 ml/y) for Group Both, 23 ml/y (SE 4 ml/y) for Group CF-only, 21 ml/y (SE 5 ml/y) for Group V-only, and 35 ml/y (SE 4 ml/y) for Group Neither during the 9-y study. The first three rates were significantly lower than that of the Group Neither after adjusting for potential confounders, with declines slower by 16 ml/y (95% CI, 9 to 23 ml/y), 12 ml/y (95% CI, 4 to 20 ml/y), and 13 ml/y (95% CI, 4 to 23 ml/y), respectively (Table 2). There was no significant difference between Group CF-only and Group V-only (Table 2). When we repeated the analysis for Group Both, Group Either (Group CF-only and Group V-only combined), and Group Neither, the *p*-values for the trend test indicated statistical significance after adjusting for potential confounders (Table S3), suggesting that improvements in both fuel type and ventilation appeared to have the greatest beneficial effect as compared to improvements in either just fuel type or ventilation.

**Table 2:** Differences in annual declines in lung function over 9

Measure	Group, Mean (SE)				Adjusted Difference between Groups, Mean (95% CI)^a^					
	Neither (*n* = 160)	CF-Only (*n* = 146)	V-Only (*n* = 89)	Both (*n* = 287)	V-Only versus Both	Neither versus Both	Neither versus V-Only	Neither versus CF-Only	CF-Only versus Both	CF-Only versus V-Only
FEV_1_ (ml/y)	35 (4)	23 (4)	21 (5)	18 (3)	3 (−6 to 11)	16 (9 to 23)	13 (4 to 23)	12 (4 to 20)	4 (−3 to 12)	2 (−8 to 11)
FVC (ml/y)	32 (4)	21 (5)	23 (6)	17 (3)	6 (−5 to 17)	16 (7 to 25)	10 (−2 to 22)	11 (1 to 21)	5 (−4 to 14)	−1 (−13 to 11)
FEV_1_/FVC ratio (percent/y)	0.2 (0.1)	0.1 (0.1)	0.0 (0.1)	0.0 (0.1)	−0.1 (−0.3 to 0.1)	0.1 (−0.1 to 0.2)	0.2 (0.0 to 0.4)	0.1 (−0.1 to 0.3)	0.0 (−0.1 to 0.2)	0.1 (−0.1 to 0.4)

a All were adjusted for the baseline lung function level for that parameter (i.e., FEV_1_, FVC, or FEV_1_/FVC ratio), age, sex, education, smoking status and intensity, ETS exposure, COPD status, BMI, occupational exposure to dust/gases/fumes, self-reported economic status, baseline biomass exposure index, the number of hours spent cooking each day, and living area size.

A dose–response relationship was also observed, i.e., the longer the period of use of improved fuel and ventilation, the slower the decline in FEV_1_ (*p*<0.05; Table 3).

**Table 3:** Differences between groups in annual declines in lung function over 9

Intervention	Participants (*n*)	FEV_1_ (ml/y)		FVC (ml/y)		FEV_1_/FVC Ratio (Percent/y)	
		Mean (SE)	Adjusted Difference	Mean (SE)	Adjusted Difference	Mean (SE)	Adjusted Difference
**Improvement in ventilation**							
0 y	315	29 (3)	8 (1 to 15)	27 (3)	7 (−1 to 15)	0.1 (0.1)	0.1 (−0.1 to 0.3)
1–4.9 y	177	20 (3)	0 (−7 to 8)	18 (4)	0 (−9 to 10)	0.1 (0.1)	0 (−0.1 to 0.2)
5–9 y	200	18 (3)	0 (reference)	19 (4)	0 (reference)	0.0 (0.1)	0 (reference)
*p*-Value		<0.001	0.026	<0.001	0.16	0.18	0.45
**Year-hours of clean fuel use for cooking**							
0 year-hours	263	30 (3)	8 (0 to 15)	28 (3)	9 (0 to 19)	0.1 (0.1)	0 (−0.1 to 0.2)
1–8.9 year-hours	262	20 (3)	1 (−7 to 8)	19 (3)	2 (−8 to 11)	0.1 (0.1)	0.1 (−0.1 to 0.2)
≥9 year-hours	157	18 (4)	0 (reference)	18 (5)	0 (reference)	0 (0.1)	0 (reference)
*p*-Value		<0.001	0.049	<0.001	0.07	0.27	0.83

All were adjusted for the baseline lung function level for that parameter (i.e., FEV_1_, FVC, or FEV_1_/FVC ratio), age, sex, education, smoking status and intensity, ETS exposure, COPD status, BMI, occupational exposure to dust/gases/fumes, self-reported economic status, baseline biomass exposure index, the number of hours spent cooking each day, and living area size.

Similar results were observed for FVC, although there was no statistically significant difference in the FEV_1_/FVC ratio between the comparison groups (Tables 2 and 3).

Further subgroup analyses revealed similar results for declines in FEV_1_ and FVC and for change in the FEV_1_/FVC ratio with regard to the effects of use of clean fuel and improved ventilation in individuals without COPD, non-smokers, and both female and male participants (Tables S4 and S5).

### Incidence of COPD

A total of 72 new cases of COPD occurred over the follow-up period among 604 participants without COPD at baseline. Compared with participants without improved ventilation for cooking, those with improvements for 5–9 y had a lower risk of COPD, with an adjusted OR of 0.39 (95% CI, 0.15 to 0.99) (Table 4). The use of both improvements had the greatest benefit for the reduction of COPD incidence, with an adjusted OR of 0.28 (95% CI, 0.11 to 0.73) (Table 4). Those who had a clean fuels index of more than 9 year-hours appeared to show a benefit over those had never used clean fuels, with an adjusted OR of 0.33 (95% CI, 0.10 to 1.03, *p* = 0.06), although the difference was not statistically significant (Table 4). There was no significant difference between the clean fuel and ventilation interventions (OR of V-only versus CF-only was 0.69 [95% CI, 0.23 to 2.08]; data not shown). We also found that the indoor pollutants PM_10_ and SO_2_ (four levels divided by quartiles of concentrations) were associated with the incidence of COPD, with adjusted relative risks of 1.92 (95% CI, 1.05 to 3.53, *p* = 0.035) and 1.87 (95% CI, 1.07 to 3.28, *p* = 0.029), respectively (Table S6). Similar results were found when COPD was identified according to the Global Initiative for Chronic Obstructive Lung Disease's diagnostic criteria as “stage 2 or worse” (FEV_1_/FVC ratio <0.70 and predicted FEV_1_<80%) (Table S7).

**Table 4:** COPD incidence and OR (95% CI) by characteristic.

Characteristic	Participants (*n* = 604)	COPD			Adjusted OR (95% CI)
		*n*	Incidence	*p*-Value	
**Use of clean fuel and improved ventilation**				0.14	
Neither	144	20	13.9%		1.00 (reference)
CF-only	129	18	14.0%		0.62 (0.23 to 1.65)
V-only	76	10	13.2%		0.43 (0.14 to 1.34)
Both	255	24	9.4%		0.28 (0.11 to 0.73)
**Cooking hours per day**				0.003	
≤1 h	220	39	17.7%		1.00 (reference)
1.1–2 h	120	10	8.3%		0.80 (0.28 to 2.31)
>2 h	264	23	8.7%		2.08 (0.86 to 5.06)
**Smoking intensity**				<0.001	
Never smoked	384	16	4.2%		1.00 (reference)
<40 pack-years	121	25	20.7%		2.10 (0.55 to 7.97)
≥40 pack-years	99	31	31.3%		3.90 (1.02 to 14.94)
**Sex**				<0.001	
Women	349	11	3.2%		1.00 (reference)
Men	255	61	23.9%		9.89 (2.25 to 43.43)
**Education**				0.58	
<6 y	540	63	11.7%		1.00 (reference)
≥6 y	64	9	14.1%		0.59 (0.21 to 1.63)
**Self-reported economic status**				0.20	
Poor	38	7	18.4%		1.00 (reference)
Not poor	566	65	11.5%		0.31 (0.09 to 1.05)
**Age group**				0.009	
40–49 y	239	18	7.5%		1.00 (reference)
50–59 y	187	21	11.2%		0.56 (0.22 to 1.40)
60–69 y	143	27	18.9%		0.85 (0.33 to 2.13)
≥70 y	35	6	17.1%		0.58 (0.14 to 2.42)
**Years of improved ventilation** a				0.053	
0 y	273	38	13.9%		1.00 (reference)
1–4.9 y	159	21	13.2%		0.52 (0.23 to 1.19)
5–9 y	172	13	7.6%		0.39 (0.15 to 0.99)
**Year-hours of clean fuel use for cooking** a				0.017	
0 year-hours	231	33	14.3%		1.00 (reference)
1–8.9 year-hours	231	31	13.4%		0.64 (0.30 to 1.36)
≥9 year-hours	142	8	5.6%		0.33 (0.10 to 1.03)

Logistic regression was used, and variables such as baseline FEV_1_/FVC, age, sex, education, self-reported economic status, smoking intensity, use of clean fuel and improved ventilation, and the number of hours spent cooking each day were entered in the model. Baseline FEV_1_/FVC was entered in the model as a continuous variable with an OR of 0.73 (95% CI, 0.68 to 0.79), and the OR of V-only versus CF-only was 0.69 (95% CI, 0.23 to 2.08).

a Entered in the model instead of the variable “use of clean fuel and improved ventilation.”

## Discussion

We found that the use of biogas instead of biomass for cooking and the improvement of kitchen ventilation were associated with a reduced decline in lung function (i.e., FEV_1_) and a reduced risk of COPD occurrence. We also demonstrated a dose–response relationship: the longer the duration with improved cooking fuels and kitchen ventilation, the greater the effects on attenuating the decline in lung function. Both interventions combined showed the greatest effect on reducing the decline in lung function and COPD incidence; either of the improvements showed a greater effect than using neither improvement.

### Strengths

To our knowledge, few studies have so far demonstrated the long-term effects of decreased indoor air pollution—through use of clean cooking fuels (mainly using biogas instead of biomass) and improved kitchen ventilation—on lung function and COPD incidence.

Chapman et al. reported that an improved coal stove was associated with reduced incidence of chronic bronchitis and emphysema based on self-reported diagnosis in a retrospective cohort study [11]. Both Romieu et al. and Smith-Sivertsen et al. reported that an improved wood stove was significantly associated with a reduced risk of respiratory symptoms in their studies in Guatemala and Mexico over 1–1.5 y [12],[13]. However, their findings differed with regard to the impact on lung function: in the study by Romieu et al, use of a Patsari stove was associated with a lower FEV_1_ decline (31 ml) compared with open fire use (62 ml) over a 1-y follow-up (*p* = 0.012) [12], but no significant effect on lung function was observed between the comparison groups in the study by Smith-Sivertsen et al. [13].

Our study showed that those who took up neither intervention (Group Neither) had a decline in FEV_1_ similar to that of current smokers as well as smokers with more than 40 pack-years, with an annual decline in FEV_1_ of approximately 35 ml per year. The decline in FEV_1_ in those who took up both interventions (Group Both) appeared to be similar to that of non-smokers, with an annual decline in FEV_1_ of approximately 18 ml per year (Tables 2, S8, and S9).

In addition, that use of improved cooking fuels and improved kitchen ventilation lowered air pollution, and that self-reported ex-smokers had ceased smoking, was confirmed in field investigations of each household kitchen and measurements of indoor air pollutants in the homes of randomly sampled participants. The diagnosis of COPD was also based on post-bronchodilator spirometry, which might minimize information bias resulting from recalling and reporting a favorable response to an intervention regardless of its physiologic efficacy.

### Limitations

Several limitations inherent in our study merit discussion. First, the participants were not randomly allocated to the study groups, which could increase the influence of confounding factors (i.e., socioeconomic status, education, occupation, smoking, and other lifestyle factors). However, we did implement some methods to minimize this confounding in the design, implementation, and analysis stages. For example, the local government financed the interventions for the entire population in the study area. To reduce potential differences in uptake due to education level, socioeconomic status, and differences in understanding of the potential benefits of the interventions, we educated and advertised to the entire population, and the local government provided partial financial aid and technical assistance for participants to solve implementation problems. As our analysis shows, there were acceptable balances between the groups classified by intervention in terms of potential confounders such as education level, occupational exposure to dust/gases/fumes, living area size per person, and economic status (Table 1). Moreover, the effect of improved ventilation and use of clean cooking fuels on lung function and COPD incidence persisted after adjusting for the above-mentioned potential confounders. Second, we conducted spirometry tests for only a portion of participants in 2005 and 2008, which might have introduced some bias; however, it did not change our conclusion that improved cooking fuels and kitchen ventilation slowed annual declines of lung function and reduced incidence of COPD, because there were consistent results when we performed analyses that included only the data from 2002 and 2011(Tables S10 and S11). Third, we estimated the change in only pre-bronchodilator FEV_1_ and FVC because a bronchodilator test was not performed among the participants without COPD at baseline. However, we made efforts to perform spirometry testing at the same time of day (i.e., in the morning) for every participant each time, and to keep the tests at comparable seasons and temperature and humidity for all participants; the post-bronchodilator spirometry tests were performed for those with pre-bronchodilator FEV_1_/FVC ratio <70%. Fourth, although participants chose improvements at different times during the observation period, our conclusion that there is a dose–response relationship between duration of use of clean cooking fuels/improved kitchen ventilation and attenuation of decline in lung function remained unchanged. Lastly, it seems unlikely, in this relatively homogenous population, that considerable bias was introduced by the fact that only one-third of sampled households underwent certain tests of indoor air pollution at the end of the study.

### Conclusions and Implications

Biomass smoke, like cigarette smoke, can increase the risk of COPD and accelerate the decline of lung function and can lead to pathological changes in patients with COPD [3]–[10],[20]–[23]. Our cohort study has confirmed, to our knowledge for the first time, that long-term interventions to improve cooking fuels (mainly using biogas instead of biomass) and kitchen ventilation were associated with improved indoor air quality, a reduced decline of lung function and reduced spirometry-measured COPD incidence.

According to World Health Organization estimates, approximately 3 billion people still rely on solid fuels, mostly in the form of biomass, for their everyday cooking and heating [24]–[27]. Biogas is an economic and clean substitute for biomass fuel for cooking. Hence, the use of biogas, an economic and clean fuel, as a substitute for biomass fuel for cooking, and improving kitchen ventilation, can possibly lead to a reduction in the global burden of COPD associated with biomass smoke, especially in non-industrialized countries. However, we recognize that implementing community interventions to change how individuals cook in rural settings in developing countries remains a challenging task. In our experience, local community efforts and local government financial and technical support have an important impact on intervention implementation. In addition, because of cooking and life habits, especially during the transitional phases of technology adoption, some households occasionally continue to use biomass fuel for specific tasks (e.g., heating water for baths in winter) depending upon fuel prices, season, and availability, even if they have good family economic conditions.

## Supporting Information

Alternative Language Abstract S1 **Chinese translation of the abstract by Yumin Zhou.**
(DOC)

Figure S1 **Intervention status of kitchen.** (A) Group Neither (neither improved ventilation nor use of clean fuels), (B) Group V-only (improved ventilation only), (C) Group CF-only (use of clean fuels only), (D) Group Both (both improved ventilation and use of clean fuels).
(TIF)

Table S1 **Characteristics of included population versus excluded population.**
(DOC)

Table S2 **Characteristics of participants by status of cooking fuel use and ventilation.**
(DOC)

Table S3 **Difference in annual decline in lung function over 9 y between indicated three groups.**
(DOC)

Table S4 **Difference in annual decline in lung function over 9 y between indicated four groups among subgroups.**
(DOC)

Table S5 **Difference in annual decline in lung function over 9 y between indicated groups among subgroups.**
(DOC)

Table S6 **Association between levels of indoor pollutants and incident cases of COPD.**
(DOC)

Table S7 **Incidence and OR (95% CI) of COPD (Global Initiative for Chronic Obstructive Lung Disease stage 2 or worse) by characteristic.**
(DOC)

Table S8 **Difference in annual decline in lung function over 9 y between groups by smoking status.**
(DOC)

Table S9 **Difference in annual decline in lung function over 9 y between groups by smoking intensity.**
(DOC)

Table S10 **Difference in annual decline in lung function over 9 y between groups by smoking intensity.**
(DOC)

Table S11 **Differences between groups in annual decline in lung function over 9 y by the history of fuel use and improvement in ventilation for cooking when the data of only 2002 and 2011 were included in analyses.**
(DOC)

## Abbreviations

BMI: body mass index
COPD: chronic obstructive pulmonary disease
ETS: environmental tobacco smoke
FEV_1_: forced expiratory volume in 1 s
FVC: forced vital capacity
OR: odds ratio
PM_10_: particulate matter with an aerodynamic diameter of 10 µm or less
SD: standard deviation
SE: standard error
