# Supplementary material for: Lung Function and Incidence of Chronic Obstructive Pulmonary Disease after Improved Cooking Fuels and Kitchen Ventilation: A 9-Year Prospective Cohort Study
Source: PLoS Med. 2014 Mar 25;11(3):e1001621. doi: 10.1371/journal.pmed.1001621 (PMC3965383; doi:10.1371/journal.pmed.1001621)
Supplement: Alternative Language Abstract S1 — Chinese translation of the abstract by Yumin Zhou. (DOC) [file pmed.1001621.s001.doc]

**烹饪燃料和厨房通风的改善对肺功能和慢性阻塞性肺疾病的影响：一项9年的前瞻性队列研究**

周玉民^1^ 邹义敏^1^ 李晓尘^1^ 陈淑云^1^ 赵祝香^1^ 何芳^1^ 邹威凤^1^ 罗秋萍^1^ 李文曦^1^ 潘仪玲^1^ 邓晓亮^1^ 王小平^2^ 丘蓉^2^ 刘世良^3^ 郑劲平^1^ 钟南山^1^ 冉丕鑫^1^*

1广州医科大学附属第一医院，广州呼吸疾病研究所，呼吸疾病国家重点实验室，广东 广州

2 韶关市第一人民医院, 广东, 韶关

3广州医科大学附属第三医院，广东 广州

通讯作者：冉丕鑫；单位：广州医科大学附属第一医院，广州呼吸疾病研究所，呼吸疾病国家重点实验室（510120）；Email：[pxran@gzhmc.edu.cn](mailto:pxran@gzhmc.edu.cn)

**研究背景：**生物燃料烟雾是慢性阻塞性肺疾病（COPD）患病的危险因素，但很少有研究阐述如何减少生物燃料烟雾相关的慢性阻塞性肺病的患病风险。本研究旨在探讨使用清洁燃料和改善厨房通风能否减少肺功能的下降及减少COPD的发病。**方法与结果：**该研究为9年的前瞻性队列研究（2002年-2011年）。来自广东的12个村庄的996名年龄40年以上的居民参与了研究。居民根据自己意愿选择改善厨房通风（如改善炉灶或厨房安装排气扇），和/或使用清洁燃料（如沼气），或继续使用以前的炉灶和生物燃料进行烹饪。在2002年和2011年对所有参与者都进行了详细问卷调查和通气功能检测，在2005年和2008年对所有参与者都进行了简易问卷调查，并对部分参与者进行了肺通气功能检测。并随机检测242例参与者厨房内空气污染物浓度（如SO2，CO，CO2，NO2和PM10）。研究评价各组参与者（仅改善厨房通风组、仅改善烹饪燃料组、烹饪燃料和厨房通风都改善组、烹饪燃料和厨房通风都没有改善组）的肺功能年下降率和慢性阻塞性肺病的新发病率。与烹饪燃料和厨房通风都没有改善组相比，仅改善烹饪燃料组和仅改善厨房通风组的第一秒用力呼气容积（ FEV1）年下降率分别减少12毫升/年（ 95 ％可信区间为4〜20毫升/年）和13毫升/年（95％可信区间为4〜23毫升/年），烹饪燃料和厨房通风都改善组的FEV1年下降减缓最明显，达到16毫升/年（ 95 ％可信区间为9〜23毫升/年）。烹饪燃料和厨房通风改善的时间越长，FEV1年下降减缓越明显（*P* <0.05）。与烹饪燃料和厨房通风都没有改善组相比，烹饪燃料和厨房通风都改善组的慢性阻塞性肺病发病风险减少，其比值比（OR）为0.28（95％可信区间为0.11〜0.73）。**结论：**使用沼气等清洁燃料代替生物燃料烹饪和改善厨房通风可能可以减少FEV1年下降率及慢性阻塞性肺病发病风险。

**试验注册号：** ChiCTR - OCH – 12002398。

**关键词**：沼气，生物燃料烟雾，干预，通风，肺功能，纵向变化，慢性阻塞性肺病
