# Supplementary figures and images for: Lung Function and Incidence of Chronic Obstructive Pulmonary Disease after Improved Cooking Fuels and Kitchen Ventilation: A 9-Year Prospective Cohort Study
Source: PLoS Med. 2014 Mar 25;11(3):e1001621. doi: 10.1371/journal.pmed.1001621 (PMC3965383; doi:10.1371/journal.pmed.1001621)

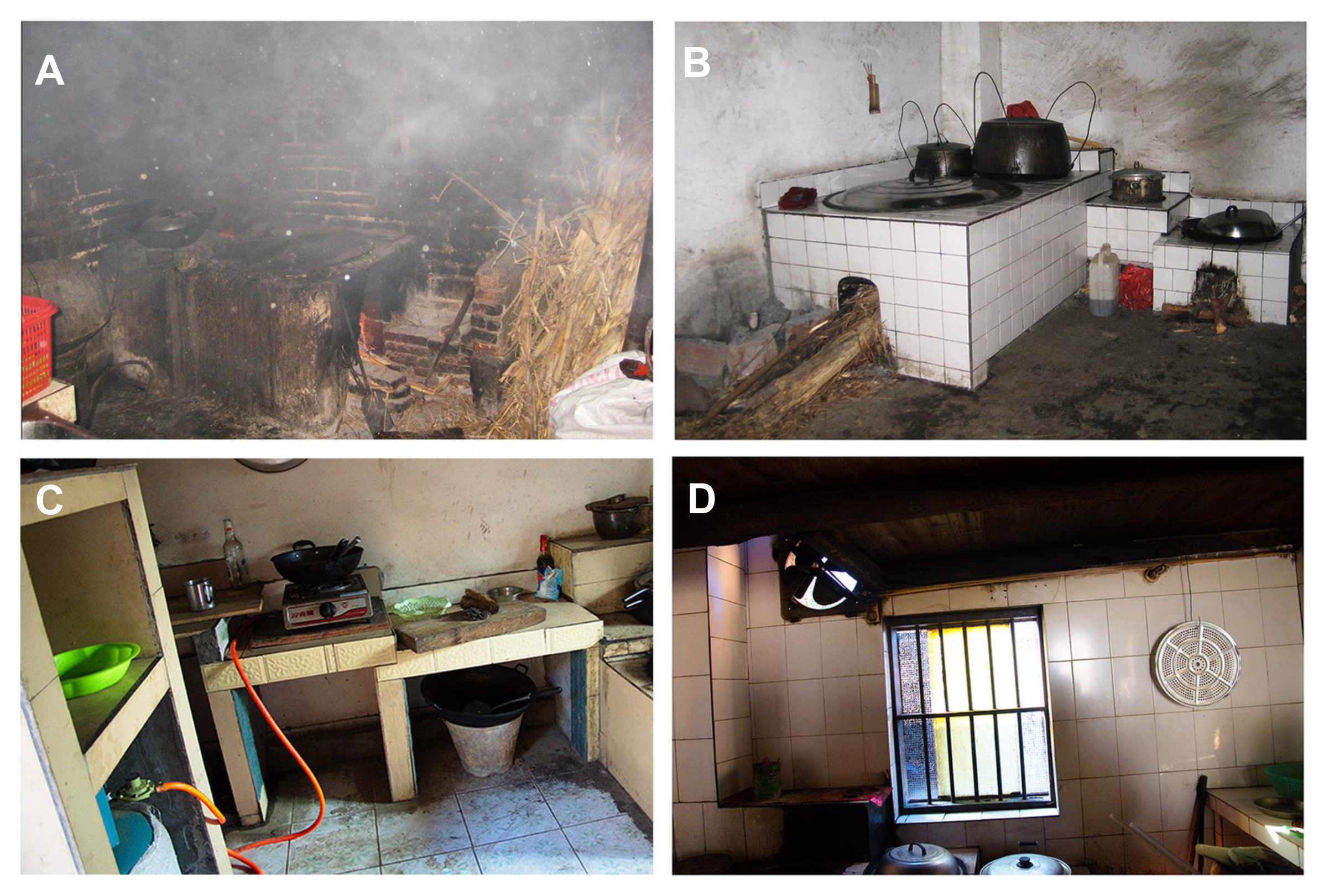

Supplement: Figure S1 — Intervention status of kitchen. (A) Group Neither (neither improved ventilation nor use of clean fuels), (B) Group V-only (improved ventilation only), (C) Group CF-only (use of clean fuels only), (D) Group Both (both improved ventilation and use of clean fuels). (TIF) [file pmed.1001621.s002.tif]
