# Supplementary material for: Lung Function and Incidence of Chronic Obstructive Pulmonary Disease after Improved Cooking Fuels and Kitchen Ventilation: A 9-Year Prospective Cohort Study
Source: PLoS Med. 2014 Mar 25;11(3):e1001621. doi: 10.1371/journal.pmed.1001621 (PMC3965383; doi:10.1371/journal.pmed.1001621)
Supplement: Table S1 — Characteristics of included population versus excluded population. (DOC) [file pmed.1001621.s003.doc]

Table S1 Characteristics of included population vs excluded population

| Characteristics | Included population  N=682 | Excluded population  N=314 | Total population  N=996 | P values |
| --- | --- | --- | --- | --- |
| Age, Mean (SD), yrs | 54.4(9.9) | 59.7(11.9) | 55.9(10.8) | <0.001 |
| Men, No. (%) | 311(45.6) | 165(52.5) | 476(47.8) | 0.041 |
| Participants educated <6 yrs, No. (%)* | 398(58.4) | 165(59.4) | 563(58.6) | 0.78 |
| COPD, No. (%) | 78(11.4) | 53(16.9) | 131(13.2) | 0.018 |
| Occupational exposure, No. (%)* | 666(97.7) | 261(93.9) | 927(96.6) | 0.004 |
| BMI, Mean (SD), kg/m2 | 22.6(2.6) | 22.4(2.9) | 22.5(2.7) | 0.37 |
| Smoking intensity, Mean (SD), pack-years *^†^ | 33.09(23.79) | 35.06(22.17) | 33.74(23.25) | 0.43 |
| Living area size, Mean (SD), m2 /persons* | 12.9(11.2) | 15.0(11.6) | 13.5(11.3) | 0.010 |
| Exposure to biomass index, Mean (SD) *‡ | 110.4(68.8) | 94.7(83.8) | 105.9(73.8) | 0.003 |
| FEV1, Mean (SD), L | 2.2(0.7) | 2.0(0.8) | 2.1(0.7) | <0.001 |
| FVC, Mean (SD), L | 2.8(0.7) | 2.6(0.8) | 2.7(0.8) | <0.001 |
| FEV1/FVC ratio, Mean (SD), % | 78.0(9.8) | 76.1(12.2) | 77.5(10.6) | 0.010 |
| Self-reported economic status^§^ |  |  |  | <0.001 |
| Poor, No. (%) | 44(6.5) | 2(0.6) | 46(4.6) |  |
| Not poor, No. (%) | 638(93.5) | 10(3.2) | 648(65.1) |  |
| Unknown, No. (%) | 0(0.0) | 301(96.2) | 301(30.3) |  |
| Current smoking status* |  |  |  | 0.001 |
| Never smoked, No. (%) | 417(61.1) | 146(52.5) | 558(58.1) |  |
| Previous smoker, No. (%) | 33(4.8) | 30(10.8) | 65(6.8) |  |
| Current smoker, No. (%) | 232(34.0) | 102(36.7) | 337(35.1) |  |
| Exposed to environmental tobacco smoke, No. (%)* | 574(84.2) | 223(80.2) | 797(83.0) | 0.14 |

*There were 36 Participants missing data.

† Calculated for smokers.

‡ Exposure to biomass index was defined as years multiplied by hours/d of exposure to biomass index for cooking at the baseline.

§There was a subject missing data.
