# Supplementary material for: Lung Function and Incidence of Chronic Obstructive Pulmonary Disease after Improved Cooking Fuels and Kitchen Ventilation: A 9-Year Prospective Cohort Study
Source: PLoS Med. 2014 Mar 25;11(3):e1001621. doi: 10.1371/journal.pmed.1001621 (PMC3965383; doi:10.1371/journal.pmed.1001621)
Supplement: Table S2 — Characteristics of participants by status of cooking fuel use and ventilation. (DOC) [file pmed.1001621.s004.doc]

**Table S2** Characteristics of participants by status of cooking fuel use and ventilation

| Characteristic | Improved Ventilation and Use of clean Fuel | | |  | Fuel use for cooking | |  | Ventilation for cooking | |
| --- | --- | --- | --- | --- | --- | --- | --- | --- | --- |
|  | Neither  (n=160) | Either  (n=235) | Both  (n=287) |  | Clean fuel  (n=433) | Polluting fuel  (n=249) |  | Improved  (n=376) | Poor  (n=306) |
| **Baseline** |  |  |  |  |  |  |  |  |  |
| Age, Mean (SD),yrs | 55.5(10.1) | 54.8(10.0) | 53.4(9.6) |  | 53.9(9.7) | 55.2(10.1) |  | 53.7(9.7) | 55.2(10.1) |
| Men, No. (%) | 75（46.9） | 111（47.2） | 125（43.6） |  | 198（45.7） | 113（45.4） |  | 163（43.4） | 148（48.4） |
| Participants educated <6 yrs, No. (%) | 150（93.8） | 208（88.5） | 253（88.2） |  | 382（88.2） | 229（92.0） |  | 332（88.3） | 279（91.2） |
| COPD, No. (%) | 16（10.0） | 30（12.8） | 32（11.1） |  | 49（11.3） | 29（11.6） |  | 45（12.0） | 33（10.8） |
| Occupational exposure, No. (%)***** | 34（21.3） | 63（26.8） | 59（20.6） |  | 99（22.9） | 57（22.9） |  | 82（21.8） | 74（24.2） |
| BMI, Mean (SD), kg/m^2^ | 22.4(2.2) | 22.4(2.7) | 22.8(2.7) |  | 22.7(2.7) | 22.3(2.4) |  | 22.6(2.7) | 22.5(2.5) |
| Smoking intensity, Mean (SD) , pack-years ^†^ | 39.1(30.4) | 32.3(21.5) | 30.1(20.5) |  | 31.2(20.3) | 36.2(28.6) |  | 30.3(21.4) | 36.2((25.9) |
| Current smoking status |  |  |  |  |  |  |  |  |  |
| Never smoked, No. (%) | 95(59.4) | 139(59.1) | 183(63.8) |  | 267(61.7) | 150(60.2) |  | 238(63.3) | 179(58.5) |
| Ex-smoker No. (%) | 5(3.1) | 17(7.2) | 11(3.8) |  | 24(5.5) | 9(3.6) |  | 15(4.0) | 18(5.9) |
| Current smoker, No. (%) | 60(37.5) | 79(33.6) | 93(32.4) |  | 142(32.8) | 90(36.1) |  | 123(32.7) | 109(35.6) |
| Exposed to environmental tobacco smoke, No. (%) | 138(86.3） | 200(85.1） | 236(82.2） |  | 337（77.9） | 184（73.9） |  | 287（76.3） | 234（76.5） |
| Self-reported poor economic status, No. (%) | 12(7.5) | 17(7.2) | 15(5.2) |  | 20(5.3) | 24(7.8) |  | 27(6.2) | 17(6.8) |
| Exposure to biomass index , Mean (SD)‡ | 116.31(69.42) | 108.78(75.13) | 108.43(62.82) |  | 108.44(66.38) | 113.81(72.82) |  | 108.63(66.83) | 112.56(71.18) |
| Living area size, Mean (SD), m^2^ /persons | 13.15(10.73) | 12.06(11.46) | 13.43(11.18) |  | 13.36(12.13) | 12.09(9.24) |  | 12.66(10.17) | 13.18(12.30) |
| FEV_1_, Mean (SD), L | 2.20(0.64) | 2.18(0.65) | 2.20(0.69) |  | 2.22（0.67） | 2.16（0.66） |  | 2.18（0.69） | 2.22（0.64） |
| % predicted FEV1, Mean (SD), % | 97.13(20.12) | 94.25(21.37) | 93.25(20.48) |  | 94.51(20.53) | 94.50(21.12) |  | 92.43(20.91) | 97.06(20.26)^※^ |
| FVC, Mean (SD), L | 2.80(0.74) | 2.81(0.73) | 2.79(0.78) |  | 2.82（0.75） | 2.77（0.75） |  | 2.78（0.77） | 2.83（0.72） |
| % predicted FVC, Mean (SD) , % | 103.71(17.54) | 102.03(18.62) | 100.07(18.13) |  | 101.50(18.42) | 101.77(17.84) |  | 99.64(18.07) | 104.00(18.09)^※^ |
| FEV_1_/FVC ratio, Mean (SD), %) | 78.33(9.38) | 77.35(10.22) | 78.42(9.69) |  | 78.26(9.75) | 77.63(9.90) |  | 77.93(9.96) | 78.15(9.62) |
| **At the end of study** |  |  |  |  |  |  |  |  |  |
| Smoking intensity, Mean (SD) , pack-years ^§^ | 47.5(31.2) | 41.7(23.5) | 39.6(22.0) |  | 40.5(21.8) | 45.3(29.9) |  | 39.9(23.2) | 44.9(27.1) |
| Current smoking status |  |  |  |  |  |  |  |  |  |
| Never smoked, No. (%) | 93（58.1） | 139（59.1） | 181（63.1） |  | 265(61.2） | 148(59.4） |  | 236(62.8） | 177(57.8） |
| Ex-smoker, No. (%) | 14（8.8） | 30（12.8） | 29（10.1） |  | 51(11.8） | 22(8.8） |  | 37(9.8） | 36(11.8） |
| Current smoker, No. (%) | 53（33.1） | 66（28.1） | 77（26.8） |  | 117(27.0） | 79(31.7） |  | 103(27.4） | 93(30.4） |
| Exposed to environmental tobacco smoke, No. (%)^＃^ | 118(75.6） | 182(77.8） | 221(78.4） |  | 337(64.7) | 184(35.3) |  | 287(55.1) | 234(44.9) |

* There were 9 Participants whose data were missing, including 3 Participants in the clean fuel group, 6 in the polluting fuel group, 2 in the improved ventilation group, 7 in the poor ventilation group, 2 in the both group, 1 in the either group and 6 in the neither group.

† calculated for 265 smokers, including 166 smokers in the clean fuel group, 99 in the polluting fuel group, 127 in the improved ventilation group, 138 in the poor ventilation group, 65 in the neither group, 96 in the either group and 104 in the both group,

‡ Baseline exposure to biomass index was defined as years multiplying hours/d of exposure to biomass index for cooking.

§calculated for 269 smokers, including 168 smokers in the clean fuel group, 101 in the polluting fuel group,129 in the improved ventilation group, 140 in the poor ventilation, 67 in the neither group, 96 in the either group and 106 in the both group.

＃ There were 10 Participants whose data were missing, including 6 Participants in the clean fuel group, 4 Participants in the polluting fuel group, 5 in the improved ventilation group, 5 in the poor ventilation group, 5 Participants in the both group, 1 Participants in the either group and 4 Participants in the neither group.

^※^ There was a significant difference between two groups.
