# Supplementary material for: Lung Function and Incidence of Chronic Obstructive Pulmonary Disease after Improved Cooking Fuels and Kitchen Ventilation: A 9-Year Prospective Cohort Study
Source: PLoS Med. 2014 Mar 25;11(3):e1001621. doi: 10.1371/journal.pmed.1001621 (PMC3965383; doi:10.1371/journal.pmed.1001621)
Supplement: Table S3 — Difference in annual decline in lung function over 9 y between indicated three groups. (DOC) [file pmed.1001621.s005.doc]

**Table S3 Difference in annual decline in lung function over 9 years between indicated three** groups

|  | Neither  (n=160) | Either  (n=235) | Both  (n=287) | Adjusted difference* | | | |
| --- | --- | --- | --- | --- | --- | --- | --- |
|  |  |  |  | Neither vs. Both | Neither vs. Either | Either vs. Both | P value |
|  | Mean(SE) | Mean(SE) | Mean(SE) | Mean (95% CI) | Mean (95% CI) | Mean (95% CI) |  |
| FEV_1_ (ml) | 35(4) | 22(3) | 18(3) | 16(9 to 23) | 14(6 to 2) | 2(-4 to 9) | <0.001 |
| FVC(ml) | 32(4) | 22 (4) | 17(3) | 15(7 to 24) | 13(4 to 22) | 2(-6 to 10) | 0.002 |
| FEV_1_/FVC ratio (%) | 0.2(0.1) | 0.0(0.1) | 0.0(0.1) | 0.1(-0.1 to 0.3) | 0.1(-0.1 to 0.3) | 0.0(-0.2to 0.1) | 0.39 |

* All were adjusted for the baseline lung function level for that parameter (i.e., FEV_1_, FVC, or FEV_1_/FVC ratio), age, sex, education, smoking status and intensity, environmental tobacco smoke, COPD status, body mass index (BMI), occupational exposure to dust/gases/fumes, baseline biomass exposure index, living area size and the number of hours spent cooking each day.
