# Supplementary material for: Lung Function and Incidence of Chronic Obstructive Pulmonary Disease after Improved Cooking Fuels and Kitchen Ventilation: A 9-Year Prospective Cohort Study
Source: PLoS Med. 2014 Mar 25;11(3):e1001621. doi: 10.1371/journal.pmed.1001621 (PMC3965383; doi:10.1371/journal.pmed.1001621)
Supplement: Table S4 — Difference in annual decline in lung function over 9 y between indicated four groups among subgroups. (DOC) [file pmed.1001621.s006.doc]

**Table S4 Difference in annual decline in lung function over 9 years between indicated four** groups among subgroups

|  | Neither | CF-only | V-only | Both | Adjusted difference between groups* | | | | | |
| --- | --- | --- | --- | --- | --- | --- | --- | --- | --- | --- |
|  |  |  |  |  | V-only vs. both | Neither vs. Both | Neither vs. V-only | Neither vs. CF-only | CF-only vs. both | CF-only vs. V-only |
|  | Mean(SE) | Mean(SE) | Mean(SE) | Mean(SE) | Mean(95%CI) | Mean(95%CI) | Mean(95%CI) | Mean(95%CI) | Mean(95%CI) | Mean(95%CI) |
| Participants without COPD (n) | 144 | 129 | 76 | 255 |  |  |  |  |  |  |
| FEV_1_ (ml/yr) | 34(4) | 20(4) | 18(5) | 17(3) | 1(-8 to 10) | 16(9 to 23) | 15(6 to 25) | 13(4 to 21) | 3(-4 to 11) | 3(-7 to 13) |
| FVC(ml/yr) | 30(4) | 15(5) | 19(6) | 15(3) | 4(-7 to 14) | 15(7 to 24) | 12(0 to 24) | 14(4 to 24) | 1(-8 to 10) | -2(-14 to 10) |
| FEV1/FVC ratio (%/yr) | 0.1(0.1) | 0.1(0.1) | -0.1(0.1) | 0.0(0.1) | -0.1(-0.3 to 0.1) | 0.1(-0.1 to 0.3) | 0.2(0.0 to 0.4) | 0.1(-0.1 to 0.2) | 0.0(-0.1 to 0.2) | 0.2(-0.1 to 0.4) |
| Participants without smoking (n) | 93 | 84 | 55 | 181 |  |  |  |  |  |  |
| FEV_1_ (ml/yr) | 29(5) | 18(5) | 15(6) | 14(3) | -1(-11 to 10) | 15(6 to 23) | 15(3 to 27) | 12(2 to 22) | 3(-6 to 12) | 3(-9 to 15) |
| FVC(ml/yr) | 33(6) | 20(6) | 22(7) | 17(4) | 3(-10 to 15) | 15(5 to 26) | 13(-1 to 27) | 13(1 to 26) | 2(-9 to 13) | -1(-15 to 14) |
| FEV1/FVC ratio (%/yr) | -0.1(0.1) | -0.1(0.1) | -0.2(0.2) | -0.1(0.1) | -0.1(-0.4 to 0.1) | 0.0(-0.2 to 0.2) | 0.1(-0.2 to 0.4) | 0.0(-0.3 to 0.2) | 0.0(-0.2 to 0.2) | 0.1(-0.1 to 0.4) |
| Women (n) | 85 | 73 | 51 | 162 |  |  |  |  |  |  |
| FEV_1_ (ml/yr) | 30(5) | 19(5) | 16(6) | 15(3) | -1(-11 to 10) | 14(5 to 22) | 14(2 to 26) | 10(0 to 21) | 3(-6 to 13) | 4(-8 to 16) |
| FVC(ml/yr) | 35(5) | 23(6) | 23(7) | 19(4) | 1(-11 to 13) | 14(4 to 25) | 13(0 to 27) | 12(0 to 24) | 2(-8 to 13) | 1(-13 to 15) |
| FEV1/FVC ratio (%/yr) | -0.1(0.1) | -0.2(0.1) | -0.3(0.2) | -0.2(0.1) | -0.1(-0.4 to 0.2) | 0.0(-0.2 to 0.2) | 0.1(-0.2 to 0.4) | 0.0(-0.3 to 0.2) | 0.0(-0.2 to 0.3) | 0.1(-0.2 to 0.4) |
| Men (n) | 75 | 73 | 38 | 125 |  |  |  |  |  |  |
| FEV_1_ (ml/yr) | 41(5) | 27(6) | 29(8) | 22(4) | 7(-8 to 21) | 21(9 to 33) | 14(-2 to 30) | 15(2 to 29) | 5(-6 to 17) | -1(-17 to 15) |
| FVC(ml/yr) | 29(7) | 19(7) | 23(10) | 15(6) | 11(-8 to 30) | 21(5 to 37) | 10(-11 to 30) | 12(-6 to 29) | 9(-6 to 24) | -2(-23 to 18) |
| FEV1/FVC ratio (%/yr) | 0.5(0.1) | 0.4(0.1) | 0.2(0.2) | 0.3(0.1) | -0.1(-0.5 to 0.2) | 0.2(-0.1 to 0.4) | 0.3(0.0 to 0.7) | 0.2(-0.1 to 0.5) | 0.0(-0.3 to 0.2) | 0.1(-0.2 to 0.5) |

CF-only: only clean fuels use; V-only: only improved ventilation; Both: improved both clean fuels and ventilation; Neither: neither improved fuels nor ventilation.

* All were adjusted for the baseline lung function level for that parameter (i.e., FEV_1_, FVC, or FEV_1_/FVC ratio), age, sex, education, smoking status and intensity, environmental tobacco smoke, COPD status, body mass index (BMI), occupational exposure to dust/gases/fumes, self-reported economic status, baseline biomass exposure index, the number of hours spent cooking each day and living area size.
