# Supplementary material for: Lung Function and Incidence of Chronic Obstructive Pulmonary Disease after Improved Cooking Fuels and Kitchen Ventilation: A 9-Year Prospective Cohort Study
Source: PLoS Med. 2014 Mar 25;11(3):e1001621. doi: 10.1371/journal.pmed.1001621 (PMC3965383; doi:10.1371/journal.pmed.1001621)
Supplement: Table S5 — Difference in annual decline in lung function over 9 y between indicated groups among subgroups. (DOC) [file pmed.1001621.s007.doc]

**Table S5 Difference in annual decline in lung function over 9 years between indicated groups among subgroups**

|  | Participants (n) | FEV_1_(ml/yr) | |  | FVC(ml/yr) | |  | FEV_1_/FVC ratio (%/yr) | |
| --- | --- | --- | --- | --- | --- | --- | --- | --- | --- |
|  |  | Mean (SE) | Adjusted difference |  | Mean (SE) | Adjusted difference |  | Mean (SE) | Adjusted difference |
| **Participants without COPD** |  |  |  |  |  |  |  |  |  |
| **Improvement on ventilation** |  |  |  |  |  |  |  |  |  |
| 0 year | 273 | 27(3) | 8(1 to 15) |  | 23(3) | 5(-4 to 13) |  | 0.1(0.1) | 0.1(0.0 to 0.3) |
| 1- 4.9year | 159 | 18(3) | 0(-7 to8) |  | 16(4) | 0(-10 to 9) |  | 0.0(0.1) | 0.0(-0.1 to 0.2) |
| 5-9 year | 172 | 16(3) | 0 (Reference) |  | 17(4) | 0 (Reference) |  | -0.1(0.1) | 0 (Reference) |
| P value |  | <0.001 | 0.033 |  | <0.001 | 0.36 |  | 0.20 | 0.22 |
| **Year-hours of clean fuel use for cooking** |  |  |  |  |  |  |  |  |  |
| 0 yr-hours | 231 | 28(3) | 6(-1 to 14) |  | 26(3) | 9(-1 to 18) |  | 0.1(0.1) | 0.0(-0.2 to 0.2) |
| 1- 8.9 yrs-hours | 231 | 17(3) | -1(-9 to 6) |  | 14(3) | -1(-10 to 8) |  | 0.0(0.1) | 0.0(-0.2 to 0.2) |
| ≥9 yr-hours | 142 | 18(4) | 0 (Reference) |  | 16(4) | 0 (Reference) |  | 0.0(0.1) | 0 (Reference) |
| P value |  | <0.001 | 0.058 |  | <0.001 | 0.046 |  | 0.68 | 0.97 |
| **Participants without smoking** |  |  |  |  |  |  |  |  |  |
| **Improvement on ventilation** |  |  |  |  |  |  |  |  |  |
| 0 year | 177 | 24(3) | 9(0 to 17) |  | 27(4) | 8(-2 to 18) |  | -0.1(0.1) | 0.0(-1.3 to 1.3) |
| 1- 4.9year | 108 | 15(4) | 2(-8 to 11) |  | 19(5) | 3(-8 to 14) |  | -0.1(0.1) | 0.1(-1.3 to 1.5) |
| 5-9 year | 128 | 14(4) | 0 (Reference) |  | 18(5) | 0 (Reference) |  | -0.2(0.1) | 0 (Reference) |
| P value |  | <0.001 | 0.09 |  | <0.001 | 0.28 |  | 0.08 | 0.81 |
| **Year-hours of clean fuel use for cooking** |  |  |  |  |  |  |  |  |  |
| 0 yr-hours | 158 | 23(4) | 4(-6 to 13) |  | 28(4) | 6(-5 to 17) |  | -0.2(0.1) | 0.1(-1.3 to 1.5) |
| 1- 8.9 yrs-hours | 149 | 14(4) | -3(-12 to 6) |  | 16(4) | -4(-15 to 7) |  | -0.1(0.1) | 0.5(-0.9 to 1.8) |
| ≥9 yr-hours | 106 | 17(4) | 0 (Reference) |  | 21(5) | 0 (Reference) |  | -0.2(0.1) | 0 (Reference) |
| P value |  | <0.001 | 0.28 |  | <0.001 | 0.14 |  | 0.08 | 0.70 |
| **Women** |  |  |  |  |  |  |  |  |  |
| **Improvement on ventilation** |  |  |  |  |  |  |  |  |  |
| 0 year | 158 | 25(4) | 9(0 to 17) |  | 29(4) | 9(-1 to18) |  | -0.2(0.1) | 0.1(-0.2 to 0.3) |
| 1- 4.9year | 99 | 16(4) | 1(-8 to 11) |  | 22(5) | 3(-8 to14) |  | -0.1(0.1) | 0.1(-0.2 to 0.3) |
| 5-9 year | 114 | 14(4) | 0 (Reference) |  | 19(5) | 0 (Reference) |  | -0.2(0.1) | 0 (Reference) |
| P value |  | <0.001 | 0.09 |  | <0.001 | 0.20 |  | 0.035 | 0.80 |
| **Year-hours of clean fuel use for cooking** |  |  |  |  |  |  |  |  |  |
| 0 yr-hours | 143 | 24(4) | 2(-7 to 11) |  | 29(4) | 3(-8 to13) |  | -0.2(0.1) | 0.0(-0.2 to 0.2) |
| 1- 8.9 yrs-hours | 130 | 15(4) | -2(-11 to7) |  | 20(4) | -4(-14 to7) |  | -0.2(0.1) | 0.1(-0.2 to 0.3) |
| ≥9 yr-hours | 98 | 18(4) | 0 (Reference) |  | 22(5) | 0 (Reference) |  | -0.2(0.1) | 0 (Reference) |
| P value |  | <0.001 | 0.55 |  | <0.001 | 0.42 |  | 0.040 | 0.84 |
| **Men** |  |  |  |  |  |  |  |  |  |
| **Improvement on ventilation** |  |  |  |  |  |  |  |  |  |
| 0 year | 148 | 34(4) | 8(-4 to 19) |  | 24(5) | 7(-8 to 22) |  | 0.4(0.1) | 0.1(-0.2 to 0.4) |
| 1- 4.9year | 78 | 25(5) | -1(-14 to 12) |  | 14(7) | -4(-21 to 13) |  | 0.3(0.1) | 0.0(-0.3 to 0.3) |
| 5-9 year | 85 | 23(5) | 0 (Reference) |  | 19(7) | 0 (Reference) |  | 0.2(0.1) | 0 (Reference) |
| P value |  | <0.001 | 0.25 |  | <0.001 | 0.33 |  | <0.001 | 0.59 |
| **Year-hours of clean fuel use for cooking** |  |  |  |  |  |  |  |  |  |
| 0 yr-hours | 120 | 37(4) | 18(5 to 32) |  | 27(6) | 21(4 to 38) |  | 0.4(0.1) | 0.1(-0.2 to 0.4) |
| 1- 8.9 yrs-hours | 132 | 26(4) | 8(-5 to 21) |  | 18(5) | 12(-6 to 29) |  | 0.4(0.1) | 0.1(-0.2 to 0.4) |
| ≥9 yr-hours | 98 | 20(6) | 0 (Reference) |  | 11(8) | 0 (Reference) |  | 0.3(0.1) | 0 (Reference) |
| P value |  | <0.0001 | 0.020 |  | <0.001 | 0.057 |  | <0.001 | 0.64 |

All were adjusted for the baseline lung function level for that parameter (i.e., FEV_1_, FVC, or FEV_1_/FVC ratio), age, sex, education, smoking status and intensity, environmental tobacco smoke, COPD status, body mass index (BMI), occupational exposure to dust/gases/fumes, baseline biomass exposure index, the number of hours spent cooking each day and living area size.
