# Supplementary material for: Lung Function and Incidence of Chronic Obstructive Pulmonary Disease after Improved Cooking Fuels and Kitchen Ventilation: A 9-Year Prospective Cohort Study
Source: PLoS Med. 2014 Mar 25;11(3):e1001621. doi: 10.1371/journal.pmed.1001621 (PMC3965383; doi:10.1371/journal.pmed.1001621)
Supplement: Table S6 — Association between levels of indoor pollutants and incident cases of COPD. (DOC) [file pmed.1001621.s008.doc]

**Table S6** Association between levels of indoor pollutants and incident cases of COPD

|  | Participants （n) * | β | S.E. | Wald values | df | P values | Adjusted RR（95% *CI*) † |
| --- | --- | --- | --- | --- | --- | --- | --- |
| SO_2_ | 212 | 0.626 | 0.286 | 4.796 | 1 | 0.029 | 1.87（1.07 to 3.28） |
| CO | 212 | 0.311 | 0.274 | 1.282 | 1 | 0.24 | 1.36（0.80 to 2.34） |
| CO_2_ | 212 | 0.127 | 0.291 | 0.189 | 1 | 0.66 | 1.14（0.64 to 2.01） |
| PM_10_ | 212 | 0.654 | 0.310 | 4.437 | 1 | 0.035 | 1.92（1.05 to 3.53） |
| NO_2_ | 212 | 0.488 | 0.290 | 2.830 | 1 | 0.09 | 1.63（0.92 to 2.88） |

Concentrations of indoor pollutants were divided four level by quartiles (<25%, 25%~50%, 50%~75% and ≥75%).

* 30 of 242 Participants were excluded out of the analysis due to being patients with COPD at the baseline.

†All were adjusted for the baseline FEV_1_/FVC ratio, age, sex, education, smoking status and intensity, environmental tobacco smoke, COPD status, body mass index (BMI), occupational exposure to dust/gases/fumes, baseline biomass exposure index, the number of hours spent cooking each day and living area size.
