# Supplementary material for: Lung Function and Incidence of Chronic Obstructive Pulmonary Disease after Improved Cooking Fuels and Kitchen Ventilation: A 9-Year Prospective Cohort Study
Source: PLoS Med. 2014 Mar 25;11(3):e1001621. doi: 10.1371/journal.pmed.1001621 (PMC3965383; doi:10.1371/journal.pmed.1001621)
Supplement: Table S7 — Incidence and OR (95% CI) of COPD (Global Initiative for Chronic Obstructive Lung Disease stage 2 or worse) by characteristic. (DOC) [file pmed.1001621.s009.doc]

**Table S7 Incidence and OR (95% CI) of COPD (Global Initiative for Chronic Obstructive Lung Disease stage 2 or worse) by characteristic**

|  | Participants  (No.=617) | COPD | | | Adjusted  OR (95% CI) |
| --- | --- | --- | --- | --- | --- |
|  |  | N | Incidence | p value |  |
| Use of clean fuel and improved ventilation |  |  |  | 0.017 |  |
| Neither | 148 | 17 | 11.5% |  | 1.00 (reference) |
| CF-only | 133 | 10 | 7.5% |  | 0.78(0.29 to 2.10) |
| V-only | 76 | 3 | 3.9% |  | 0.36(0.09 to 1.45) |
| Both | 260 | 10 | 3.8% |  | 0.33(0.12 to 0.87) |
| Cooking hours per day |  |  |  | 0.22 |  |
| ≤1 hour | 227 | 20 | 8.8% |  | 1.00 (reference) |
| 1.1 - 2 hours | 122 | 4 | 3.3% |  | 0.48(0.12 to 1.96) |
| > 2 hours | 268 | 16 | 6.0% |  | 1.41(0.56 to 3.57) |
| Smoking intensity |  |  |  | <0.001 |  |
| Never smoked | 390 | 12 | 3.1% |  | 1.00 (reference) |
| <40 pack-yrs | 122 | 8 | 6.6% |  | 2.21(0.37 to13.07) |
| ≥40 pack-yrs | 105 | 20 | 19.0% |  | 5.85(1.06 to 32.14) |
| Current smoking status |  |  | % | <0.001 |  |
| Current smoker | 172 | 25 | 14.5% |  | 1.00 (reference) |
| Ex-smoker | 55 | 3 | 5.5% |  | 0.26(0.06 to1.18) |
| Sex |  |  |  | <0.001 |  |
| Women | 354 | 10 | 2.8% |  | 1.00 (reference) |
| Men | 263 | 30 | 11.4% |  | 1.69(0.0.28 to 10.14) |
| Age group |  |  |  | 0.011 |  |
| 40-49 yrs | 241 | 9 | 3.7% |  | 0.68(0.23 to 2.08) |
| 50-59 yrs | 191 | 11 | 5.8% |  | 0.95(0.23 to 2.73) |
| 60-69 yrs | 150 | 18 | 12.0% | <0.001 | 0.73(0.12 to 4.36) |
| ≥70 yrs | 35 | 2 | 5.7% |  | 1.00 (reference) |
| Education |  |  |  | 0.60 |  |
| <6yrs | 553 | 36 | 6.5% |  | 1.00 (reference) |
| ≥6yrs | 64 | 4 | 6.3% |  | 0.75(0.20 to 2.82) |
| Self-reported economy status |  |  |  | 0.037 |  |
| Poor | 40 | 6 | 15.0% |  | 1.00 (reference) |
| Not poor | 577 | 34 | 5.9% |  | 0.29(0.09 to 0.93) |
| Improved ventilation^*^ |  |  |  | 0.002 |  |
| 0 yr | 281 | 27 | 9.6% |  | 1.00 (reference) |
| 1-4.9 yrs | 162 | 9 | 5.6% |  | 0.46(0.18 to 1.18) |
| 5-9 yrs | 174 | 4 | 2.3% |  | 0.28(0.08 to 0.95) |
| Year-hours of clean fuel use for cooking^*^ |  |  |  | 0.018 |  |
| 0 yr-hours | 235 | 22 | 9.4% |  | 1.00 (reference) |
| 1- 8.9 yrs-hours | 237 | 13 | 5.5% |  | 0.85(0.35 to 2.05) |
| ≥9 yr-hours | 145 | 5 | 3.4% |  | 0.62(0.18 to 2.09) |

Forward stepwise logistic regression was used and variables such as baseline FEV_1_/FVC, smoking status and intensity, self-reported economic status, use of clean fuel and improved ventilation and the number of hours spent cooking each day were entered in the final model. Baseline FEV_1_/FVC was entered in the model as a continuous variable with an OR of 0.81(95% CI, 0.76 to 0.87).

*entered in the model instead of the variable “use of clean fuel and improved ventilation”.
