# Supplementary material for: Lung Function and Incidence of Chronic Obstructive Pulmonary Disease after Improved Cooking Fuels and Kitchen Ventilation: A 9-Year Prospective Cohort Study
Source: PLoS Med. 2014 Mar 25;11(3):e1001621. doi: 10.1371/journal.pmed.1001621 (PMC3965383; doi:10.1371/journal.pmed.1001621)
Supplement: Table S8 — Difference in annual decline in lung function over 9 y between groups by smoking status. (DOC) [file pmed.1001621.s010.doc]

**Table S8 Difference in annual decline in lung function over 9 years between groups by smoking status**

|  | Current  smoker | |  | Ex-smoker | |  | Never  smoked | |  | Adjusted difference | | | | | |
| --- | --- | --- | --- | --- | --- | --- | --- | --- | --- | --- | --- | --- | --- | --- | --- |
|  |  |  |  |  |  |  |  |  |  | Current vs. Never | | Ex-smoker vs. Never | | Current vs. Ex-smoker | |
|  | n | Mean  (SE) |  | n | Mean  (SE) |  | n | Mean  (SE) |  | Mean  (95% CI) | P value | Mean  (95% CI) | P value | Mean  (95% CI) | P value |
| Men and women combined* |  |  |  |  |  |  |  |  |  |  |  |  |  |  |  |
| FEV1 (ml) | 196 | 34(3) |  | 73 | 25(5) |  | 413 | 18(2) |  | 19(7 to 32) | 0.0028 | 7(-8 to 22) | 0.3769 | 13(2 to 23) | 0.0153 |
| FVC(ml) | 196 | 24(4) |  | 73 | 20(7) |  | 413 | 22(3) |  | 17(1 to 33) | 0.0350 | 7(-12 to 25) | 0.4935 | 10(-2 to 23) | 0.11 |
| FEV1/FVC ratio (%) | 196 | 0.4(0.1) |  | 73 | 0.3(0.1) |  | 413 | -0.1(0.1) |  | 0.4(0.1 to 0.7) | 0.0146 | 0.2(-0.1 to 0.6) | 0.2164 | 0.1(-0.1 to 0.4) | 0.22 |
| Men only |  |  |  |  |  |  |  |  |  |  |  |  |  |  |  |
| FEV1 (ml) | 195 | 34(3) |  | 72 | 24(6) |  | 44 | 15(7) |  | 22(7 to 37) | 0.0035 | 8(-10 to 25) | 0.3961 | 15(3 to 26) | 0.0162 |
| FVC(ml) | 195 | 24(4) |  | 72 | 20(7) |  | 44 | 6(9) |  | 20(1 to 40) | 0.0377 | 7(-16 to 30) | 0.5686 | 14(-2 to 29) | 0.08 |
| FEV1/FVC ratio (%) | 195 | 0.4(0.1) |  | 72 | 0.3(0.1) |  | 44 | 0.1(0.2) |  | 0.4(0.1 to 0.7) | 0.0160 | 0.2(-0.1 to 0.6) | 0.2269 | 0.2(-0.1 to 0.4) | 0.22 |

*Only one female subject has ever smoked.

All were adjusted for the baseline lung function level for that parameter (i.e., FEV_1_, FVC, or FEV_1_/FVC ratio), age, sex, education, improved fuels and kitchen ventilation for cooking, environmental tobacco smoke, COPD status, body mass index (BMI), occupational exposure to dust/gases/fumes, baseline biomass exposure index, self-reported economic status, the number of hours spent cooking each day and living area size.
