# Supplementary material for: Lung Function and Incidence of Chronic Obstructive Pulmonary Disease after Improved Cooking Fuels and Kitchen Ventilation: A 9-Year Prospective Cohort Study
Source: PLoS Med. 2014 Mar 25;11(3):e1001621. doi: 10.1371/journal.pmed.1001621 (PMC3965383; doi:10.1371/journal.pmed.1001621)
Supplement: Table S9 — Difference in annual decline in lung function over 9 y between groups by smoking intensity. (DOC) [file pmed.1001621.s011.doc]

**Table S9 Difference in annual decline in lung function over 9 years between groups by smoking intensity**

|  | Participants (n) | FEV_1_ (ml/yr) | |  | FVC (ml/yr) | |  | FEV_1_/FVC ratio (%/yr) | |
| --- | --- | --- | --- | --- | --- | --- | --- | --- | --- |
|  |  | Mean (SE) | Adjusted difference |  | Mean (SE) | Adjusted difference |  | Mean (SE) | Adjusted difference |
| Men and women combined* |  |  |  |  |  |  |  |  |  |
| 0 pack-yr | 413 | 18(2) | 0 (Reference) |  | 22(3) | 0 (Reference) |  | -0.1(0.1) | 0 (Reference) |
| 1- 39.9 pack-yrs | 142 | 29(4) | 14(1 to 26) |  | 20(5) | 11(-4 to 26) |  | 0.4(0.1) | 0.3(0.0 to 0.6) |
| ≥40 pack-yrs | 127 | 34(4) | 17(5 to 30) |  | 26(5) | 15(0 to 31) |  | 0.4(0.1) | 0.3(0.1 to 0.6) |
| P value |  | <0.001 | 0.0263 |  | <0.001 | 0.16 |  | <0.001 | 0.06 |
| Men only |  |  |  |  |  |  |  |  |  |
| 0 pack-yr | 44 | 15(7) | 0 (Reference) |  | 6(9) | 0 (Reference) |  | 0.1(0.2) | 0 (Reference) |
| 1- 39.9 pack-yrs | 140 | 28(4) | 16(2 to 31) |  | 20(5) | 13(-5 to 32) |  | 0.4(0.1) | 0.4(0.0 to 0.7) |
| ≥40 pack-yrs | 127 | 34(4) | 20(5 to 35) |  | 26(6) | 18(-1 to 37) |  | 0.4(0.1) | 0.4(0.1 to 0.7) |
| P value |  | <0.001 | 0.0303 |  | <0.001 | 0.18 |  | <0.001 | 0.05 |

*Only one female subject has ever smoked.

All were adjusted for the baseline lung function level for that parameter (i.e., FEV_1_, FVC, or FEV_1_/FVC ratio), age, sex, education, improved fuels and kitchen ventilation for cooking, environmental tobacco smoke, COPD status, body mass index (BMI), occupational exposure to dust/gases/fumes, baseline biomass exposure index, self-reported economic status, the number of hours spent cooking each day and living area size.
