# Supplementary material for: Lung Function and Incidence of Chronic Obstructive Pulmonary Disease after Improved Cooking Fuels and Kitchen Ventilation: A 9-Year Prospective Cohort Study
Source: PLoS Med. 2014 Mar 25;11(3):e1001621. doi: 10.1371/journal.pmed.1001621 (PMC3965383; doi:10.1371/journal.pmed.1001621)
Supplement: Table S10 — Difference in annual decline in lung function over 9 y between groups by smoking intensity. (DOC) [file pmed.1001621.s012.doc]

**Table S10 Differences in annual declines in lung function over 9 years between the indicated groups when the data of only 2002 and 2011were included in analyses**

|  | Neither  (n=160) | CF-only  (n=146) | V-only  (n=89) | Both  (n=287) | Adjusted difference between groups* | | | | | |
| --- | --- | --- | --- | --- | --- | --- | --- | --- | --- | --- |
|  |  |  |  |  | V-only vs. both | Neither vs. Both | Neither vs. V-only | Neither vs. CF-only | CF-only vs. both | CF-only vs. V-only |
|  | Mean(SE) | Mean(SE) | Mean(SE) | Mean(SE) | Mean (95% CI) | Mean (95% CI) | Mean (95% CI) | Mean (95% CI) | Mean (95% CI) | Mean (95% CI) |
| FEV_1_ (ml/yr) | 35(3) | 23(3) | 21(4) | 18(2) | 3(-5 to 11) | 17(10 to 23) | 13(5 to 22) | 12(4 to 20) | 5(-2 to 11) | 1(-7 to 10) |
| FVC (ml/yr) | 32(3) | 21(4) | 23(5) | 17(3) | 7(-3 to 17) | 16(8 to 25) | 9(-2 to 20) | 11(2 to 21) | 5(-3 to 14) | -2(-13 to 9) |
| FEV_1_/FVC ratio (%/yr) | 0.2(0.1) | 0.1(0.1) | 0.0(0.1) | 0.0(0.0) | -0.1(-0.3 to 0.0) | 0.1(-0.1 to 0.2) | 0.2(0.0 to 0.4) | 0.1(-0.1 to 0.3) | 0.0(-0.1 to 0.2) | 0.1(-0.1 to 0.3) |

* All were adjusted for the baseline lung function level for that parameter (i.e., FEV_1_, FVC, or FEV_1_/FVC ratio), age, sex, education, smoking status and intensity, environmental tobacco smoke, COPD status, body mass index (BMI), occupational exposure to dust/gases/fumes, self-reported economic status, baseline biomass exposure index, the number of hours spent cooking each day and living area size.
