# Supplementary material for: Lung Function and Incidence of Chronic Obstructive Pulmonary Disease after Improved Cooking Fuels and Kitchen Ventilation: A 9-Year Prospective Cohort Study
Source: PLoS Med. 2014 Mar 25;11(3):e1001621. doi: 10.1371/journal.pmed.1001621 (PMC3965383; doi:10.1371/journal.pmed.1001621)
Supplement: Table S11 — Differences between groups in annual decline in lung function over 9 y by the history of fuel use and improvement in ventilation for cooking when the data of only 2002 and 2011 were included in analyses. (DOC) [file pmed.1001621.s013.doc]

**Table S11 Differences between groups in annual decline in lung function over 9 years by the history of fuel use and improvement in ventilation for cooking when the data of only 2002 and 2011were included in analyses**

|  | Participants (n) | FEV_1_ (ml/yr) | |  | FVC (ml/yr) | |  | FEV_1_/FVC ratio (%/yr) | |
| --- | --- | --- | --- | --- | --- | --- | --- | --- | --- |
|  |  | Mean (SE) | Adjusted difference |  | Mean (SE) | Adjusted difference |  | Mean (SE) | Adjusted difference |
| **Improvement in ventilation** |  |  |  |  |  |  |  |  |  |
| 0 yr | 315 | 29(2) | 8(2 to 14) |  | 27(2) | 7(-2 to 15) |  | 0.1(0.0) | 0.1(0.0 to 0.3) |
| 1- 4.9 yrs | 177 | 20(3) | 0(-7 to 8) |  | 18(3) | 0(-9 to 10) |  | 0.1(0.1) | 0 (-0.1 to 0.2) |
| 5-9 yrs | 200 | 18(2) | 0 (Reference) |  | 19(3) | 0 (Reference) |  | 0.0(0.1) | 0 (Reference) |
| P value |  | <0.001 | 0.018 |  | <0.001 | 0.21 |  | 0.020 | 0.33 |
| **Year-hours of clean fuel use for cooking** |  |  |  |  |  |  |  |  |  |
| 0 yr-hours | 263 | 30(2) | 8(1 to 16) |  | 28(3) | 10(1 to 20) |  | 0.1(0.0) | 0.1 (-0.1 to 0.2) |
| 1- 8.9 yrs-hours | 262 | 20(2) | 1(-6 to 8) |  | 19(3) | 2(-7 to 12) |  | 0.1(0.0) | 0.0(-0.1 to 0.2) |
| ≥9 yr-hours | 157 | 18(3) | 0 (Reference) |  | 18(3) | 0 (Reference) |  | 0.0(0.1) | 0 (Reference) |
| P value |  | <0.001 | 0.023 |  | <0.001 | 0.06 |  | 0.049 | 0.88 |

All were adjusted for the baseline lung function level for that parameter (i.e., FEV_1_, FVC, or FEV_1_/FVC ratio), age, sex, education, smoking status and intensity, environmental tobacco smoke, COPD status, body mass index (BMI), occupational exposure to dust/gases/fumes, self-reported economic status, baseline biomass exposure index, the number of hours spent cooking each day and living area size.
